# 

**Published:** 1884-05-20

**Authors:** 


### TABLE OF CONTENTS.

ORIGINAL AND SELECTED ARTICLES:

A Diagnostic Sign of Tuberculous Meningitis, by E. Van Goitdsnoven, M. D., of Georgia, 161. Removal of Foreign Bodies From the Surface of the Eye and Lids, by S. L. Frank, M. D., 163 Spermatorrhœa, by J. S. Geigley, M. D., Lewiston, Ill., 166. Scarlet Fever and Measles in Dogs and Cats, by John C. Peters, M. D., New York, 170. Detroit Medical and Library Association, 173. Ergot in Whooping Cough, by John Dewar, L. R. C. P. Etc., London, England, 176. Phytollacca Decandra and Mint in Inflamed Mammæ, 177.

ABSTRACTS AND GLEANINGS:

California Laurel, 178. The Bromide of Sodium vs. Like Salts of Potassium, 179. Tonga; Tincture of Gelsemium Hypodermically in Convulsions, 180. Chancres and Old Sores; Incubation of Infants, 181. A New Method of Removing Nasal Polypi; How to Take a Pill, 182. Bromide of Arsenic in Diabetes; Investigations into the Physiological and Therapeutic Properties of Trinotrine, 183. Mastitis, 184. Experiments With the Sputa of Phthisical Patients; Common Sense in the Lying-in Room, 185. Santonine; Administering Medicines, 186. Bed Pan in Child-bed, 187. Treatment of Placenta Prævia; Renal Calculus, 188. Lobelia, 189. A Bird's Appetite; Radical Cure of Hemorrhoids; Remarkable Recovery; Digitalis as an Anaphrodisiac, 190.

SCIENTIFIC ITEMS:

Mending a Submarine Cable; Achromatic Microscope, 191. Carbolic Acid and the Devil; Pressure in a Diving-bell, 192.

PRACTICAL NOTES AND FORMULÆ.

Carbolic Acid Injections in Hæmorrhoids; Glycerine in Febrile Conditions, 193. Scarlatina; Aperient Mixture (Alkaline Mixture of Aloes); Emulsion of Copaiba; Tolu Rock and Rye, 194. Spermatorrhœa; Dry Seborrhœa of the Scalp; A New Chewing Gum; Congestive Headache—Nickel; Disguising the Taste of Tincture of Iron, 195.

EDITORIALS AND MISCELLANEOUS.

American Medical Association, 196. Georgia Medical Association; The People and the Code; A Liberal Doctor, 197. Daily Star and Herald (Panama); Books and Pamphlets Received, 198. Receipted; Special Notices, 200.

